# Identification of distinct immune landscapes using an automated nine-color multiplex immunofluorescence staining panel and image analysis in paraffin tumor tissues

**DOI:** 10.1038/s41598-021-83858-x

**Published:** 2021-02-25

**Authors:** Edwin R. Parra, Jie Zhai, Auriole Tamegnon, Nicolas Zhou, Renganayaki Krishna Pandurengan, Carmelia Barreto, Mei Jiang, David C. Rice, Caitlin Creasy, Ara A. Vaporciyan, Wayne L. Hofstetter, Anne S. Tsao, Ignacio I. Wistuba, Boris Sepesi, Cara Haymaker

**Affiliations:** 1 Department of Translational Molecular Pathology, The University of Texas MD Anderson Cancer Center, Unit 9512130 Holcombe Blvd, Houston, TX 77030 USA; 2 Departments of Thoracic and Cardiovascular Surgery, The University of Texas MD Anderson Cancer Center, Houston, TX USA; 3 Melanoma Medical Oncology, The University of Texas MD Anderson Cancer Center, Houston, TX USA; 4 Thoracic/Head and Neck Medical Oncology, The University of Texas MD Anderson Cancer Center, Houston, TX USA; 5 Division of Cancer Medicine, The University of Texas MD Anderson Cancer Center, Houston, TX USA

**Keywords:** Cancer, Immunology, Biomarkers, Pathogenesis

## Abstract

Immune profiling is becoming a vital tool for identifying predictive and prognostic markers for translational studies. The study of the tumor microenvironment (TME) in paraffin tumor tissues such as malignant pleural mesothelioma (MPM) could yield insights to actionable targets to improve patient outcome. Here, we optimized and tested a new immune-profiling method to characterize immune cell phenotypes in paraffin tissues and explore the co-localization and spatial distribution between the immune cells within the TME and the stromal or tumor compartments. Tonsil tissues and tissue microarray (TMA) were used to optimize an automated nine-color multiplex immunofluorescence (mIF) panel to study the TME using eight antibodies: PD-L1, PD-1, CD3, CD8, Foxp3, CD68, KI67, and pancytokeratin. To explore the potential role of the cells into the TME with this mIF panel we applied this panel in twelve MPM cases to assess the multiple cell phenotypes obtained from the image analysis and well as their spatial distribution in this cohort. We successful optimized and applied an automated nine-color mIF panel to explore a small set of MPM cases. Image analysis showed a high degree of cell phenotype diversity with immunosuppression patterns in the TME of the MPM cases. Mapping the geographic cell phenotype distribution in the TME, we were able to identify two distinct, complex immune landscapes characterized by specific patterns of cellular distribution as well as cell phenotype interactions with malignant cells. Successful we showed the optimization and reproducibility of our mIF panel and their incorporation for comprehensive TME immune profiling into translational studies that could refine our ability to correlate immunologic phenotypes with specific patterns of cells distribution and distance analysis. Overall, this will improve our ability to understand the behavior of cells within the TME and predict new treatment strategies to improve patient outcome.

## Introduction

Harnessing the immune response through immunotherapy approaches has revolutionized the treatment for patients with cancer ^1^. Immune checkpoints such as programmed cell death protein 1 (PD-1) and one of its ligands, programmed cell death ligand 1 (PD-L1), mainly exist on immune cells and inhibit T cell responses by promoting their apoptosis. Some malignancies take advantage of this immune checkpoint pathway by expressing PD-L1 on their cell surfaces regulated by the tumor microenvironment (TME) and offering a mechanism to evade the immune response^2–4^. Because immune checkpoint inhibitors (anti–PD-1/PD-L1) have been clinically successful, research efforts are focusing on characterizing the TME in cancer patients to reveal the presence of distinct immunologic phenotypes to identify mechanisms of resistance as well as new, actionable targets to improve patient outcome^5–7^.

Malignant pleural mesothelioma (MPM) is a rare type of cancer, strongly attributable to exposure to carcinogenic mineral fibers^8, 9^. Men are at a higher risk than women, likely due to higher occupational exposure^10^. Traditional treatment strategies, including surgery, chemotherapy, and radiotherapy, can be delivered separately or as part of multimodality treatment; however, patient prognosis remains poor despite these treatment modalities^11^. MPM is characterized by a complex immune landscape, which is modulated by the TME, but also has great heterogeneity^12^. This heterogeneity might predict different prognoses^13–15^. A deeper understanding of this complex, yet heterogeneous immune environment in rare tumors such as MPM could inform on the cellular interactions that may promote immune suppression and tumor growth. These observations can result in predictive biomarkers that can guide the identification of new, actionable immunotherapeutic interventions^16^. Tumor-associated immune cells (TAICs) can be directly targeted through both antagonistic and agonistic antibody-based immunotherapy approaches^17–19^. Multiplex immunofluorescence (mIF) staining of immune system–based biomarkers can help to characterize the TME, and we previously showed that distinct immunologic phenotypes can exist in the same tissue section in formalin-fixed and paraffin-embedded (FFPE) tumor tissues^20–22^. Using multiplex immunofluorescence we have also the ability to study the spatial distribution between cell populations to correlate with clinical outcomes and enhance the convenience of immune profiling^23^.

In the current study, our aim was optimizing an automated nine-color mIF panel with tyramide signal amplification and a multispectral image analysis system for TME immune profiling of FFPE tumor tissues. We applied this mIF panel to a small cohort of MPM samples to explore the PD-L1/PD-1 axis, cellular phenotype proliferation in the TME and their geographic distribution and spatial proximity of cell phenotypes related to malignant cells to show the potential capability of the data obtained through this methodology for immune profiling FFPE tumor tissues in translational studies.

## Methods

### FFPE tissue specimens for panel optimization and mesothelioma cohort

Sequential 4-µm-thick sections from human reactive tonsil FFPE tissues were prepared for conventional immunohistochemistry (IHC) and for single immunofluorescence (IF) and mIF optimization as previously described^24^. Additionally, 4-µm-thick sections from representative MPM FFPE block cases (N = 12 cases), randomly selected from our archived tissue bank, were prepared for staining purpose of the mIF panel and exploratory analysis with this mIF panel. In parallel tissue microarray (TMA) was created using three 1.0-mm tissue cores obtained from the same MPM FFPE blocks to see the consistency and reproducibility of the mIF staining panel across the time.

Additionally, all the available clinicopathologic information was retrieved from the electronic clinical records for those patients (Supplementary Table 1) to show the characteristic of the cohort for exploratory purposes; this included age, sex, smoking history, asbestos exposure, lymph node status, pathologic TNM stage, receipt of neoadjuvant and adjuvant treatment, and follow-up information for recurrence and vital status.

This study was approved by The University of Texas MD Anderson Cancer Center institutional review board and all methods were performed in accordance with the relevant guidelines and regulations.

### Immunohistochemistry marker optimization

Chromogen-based IHC analysis was performed by using an automated staining system (BOND-MAX; Leica Biosystems, Vista, CA) with antibodies against the following: pancytokeratin (epithelial cell marker; clone AE1/AE3, dilution 1:300; Dako, Carpinteria, CA), PD-L1 (clone E1L3N, dilution 1:100; Cell Signaling Technology, Danvers, MA), CD8 (cytotoxic T-cell marker; clone C8/144B, dilution 1:20; Thermo Fisher Scientific, Waltham, MA), CD3 (T-cell lymphocyte marker; clone D7A6E, dilution 1:100; Dako), PD-1 (clone EPR4877-2, dilution 1:250; Abcam, Cambridge, MA), Foxp3 (regulatory T-cell marker; clone 206D, dilution 1:50; BioLegend, San Diego, CA), KI67 (proliferation marker; clone MIB-1, dilution 1:100; Agilent Technologies, Santa Clara, CA), and CD68 (macrophage marker; clone PG-M1, dilution 1:450; Dako). Expression of all cell markers was detected using a Novocastra Bond Polymer Refine Detection Kit (catalog #DS9800; Leica Biosystems) with a diaminobenzidine reaction to detect antibody labelling and hematoxylin counterstaining. To guarantee specificity and sensitivity of the different antibodies, several tests were done until we obtained a reproducible pattern and correct geographical distribution of the different antibodies in the control tissues (Supplementary Fig. 1).

### Single IF antibody optimization

After chromogen-based IHC optimization, all the markers were assessed by single IF staining to optimize the antibodies. Single IF staining was performed automatically using the Leica Bond RX (Leica Biosystems), the fluorophores contained in the Opal 7 kit (catalog #NEL797001KT; Akoya Biosciences, Waltham, MA)—4′,6-diamidino-2-phenylindole (DAPI), Opal Polaris 520, Opal Polaris 540, Opal Polaris 570, Opal Polaris 620, Opal Polaris 650, and Opal Polaris 690—and the individual tyramide signal amplification fluorophores Opal Polaris 480 (catalog #FP1500001KT) and Opal Polaris 780 kit (catalog #FP1501001KT, Akoya Biosciences).

Each of the fluorophores linked to one antibody to detect various targets proposed in the mIF panel. After baking and dewaxing (Bond Dewax Solution, Leica Biosystems), the slides were heated at 95 °C for 20 min with Bond Antigen Retrieval Tris–EDTA buffer (for PD-1 and KI67) or citrate buffer (for the remaining markers), to open antibody epitopes. The slides were then incubated between 30 min and 1 h (depending on which antibody was used at room temperature) with the same primary antibodies used for IHC staining against the immune markers at similar dilutions: CD3 (dilution 1:100), CD8 (dilution 1:25), PD-1 (dilution 1:250), PD-L1 (dilution 1:1500), Foxp3 (dilution 1:50), KI67 (dilution 1:100), CD68 (dilution 1:50), and panCK (dilution 1:100). The slides were washed 3 times with 1 × 2-methyl-2H-isothiazol-3-one (Bond Wash Solution, catalog #AR9590, Leica Biosystems), then incubated for 10 min at room temperature with polymer horseradish peroxidase (HRP), following anti-mouse or anti-rabbit secondary antibodies (Akoya Biosciences). After successive washes, around 5 times, with Bond Wash Solution, the slides were incubated for 10 min with one of the following fluorophore tyramides to detect antibody staining, prepared according to the manufacturer’s instructions: Opal Polaris 480, Opal Polaris 520, Opal Polaris 540, Opal Polaris 570, Opal Polaris 620, Opal Polaris 650, Opal Polaris 690, Opal Polaris 780 (dilution 1:50 to 1:150). After 4 additional washes with Bond Wash Solution, the slides were counterstained with DAPI for 5 min. The slides were taken out of the autostainers and manually mounted with ProLong Diamond Antifade Mountant (Invitrogen/Thermo Fisher Scientific, Waltham, MA). For Opal 780, an additional step was needed; after the HRP and washes, the slides were washed with the Opal TSA-Dig reagent 1 time followed by 10 min of incubation with Opal TSA-Dig reagent. After 1 wash with Bond Wash Solution, the slides were incubated with citrate buffer Bond Antigen Retrieval at 95 °C for 20 min, followed by 4 washes with Bond Wash Solution and incubation with Opal Polaris 780 reagent for 1 h. Finally, the slides were washed 3 times with Bond Wash Solution to finalize with the DAPI staining for 5 min as explained above.

Using the automated protocol, the sequence of the antibodies in the panel was set up to obtain the same dynamic ranges from the different antibodies linked with their particular fluorophore to obtain a similar range of expression between 50 to 150 ns of exposure time using the Vectra/Polaris 3.0.3 scanner system (Akoya Biosciences) for each antibody expression in the panel. This procedure is used to avoid cross-talking reaction between the fluorophores or blocking of the expression of one antibody by another when it was expressed in the same cell compartment (umbrella effect)^25^.

For each run of staining, three types of autofluorescence (negative control) slides were run in parallel as follows: (1) using the primary and secondary antibodies and omitting the fluorophore tyramides, (2) using the fluorophore tyramides and secondary antibodies and omitting the primary antibodies, and (3) using only the secondary antibodies and omitting the fluorophore tyramides and primary antibodies to extract endogenous and exogenous autofluorescence from the tissues during the image analysis of the tissues.

### Spectral library

In parallel, the spectral library was created for multispectral image analysis visualization extraction. Using an antibody with higher amount of positive cells in tonsils as CD20 antibody (B-cell marker; clone L26, dilution 1:100; Dako), the eight fluorophore tyramides were stained using similar conditions as for the single IF protocol without DAPI while trying to maintain similar range of expression (Supplementary Fig. 2).

### Multiplex IF optimization

Once each target was optimized in single protocols, they were combined to create the multiplex protocol to generate the stained slides. We applied primary antibodies to different tonsil specimens as positive controls to optimize the concentrations previously determined on the single IF staining. Staining was performed consecutively by using the same steps as those used in single IF in the Leica Bond RX autostainer, and the detection for each marker was completed before application of the next antibody. The automated machine was set up with the best sequence of each antibody combined with an Opal fluorophore tyramide to achieve the multiplex staining panel (Supplementary Table 2). A negative control for each run was also included as described for the single IF staining. Several times the staining was respited to guarantee a consistent staining pattern in the positive controls.

### Staining consistency using TMA

To assess the consistency of the staining markers with the mIF panel over time the TMA was staining with the on consecutive sections at three time points with a 1-week interval (week 1, week 2 and week 3), the 36 cores contained in the TMA from the 12 MPM cases were stained in the autostainer Leica Bond RX and scanned at the three time points using the Vectra/Polaris 3.0.3 multispectral imaging system (Akoya Biosciences) through the full emission spectrum from 440 to 780 nm, to extract fluorescence intensity information from the images using positive tonsil controls from each run staining to calibrate the spectral image scanner protocol at 20 × magnification (0.5 µm/pixel). To determine consistence of the staining across the time each marker was quantify individually using a spectral signature for each fluorophore obtained by the “spectral unmixing library” using the the same algorithm from the InForm 2.4.8 image analysis software (Akoya Biosciences). The percentages representing for each marker were calculated by dividing the absolute number of each marker by the absolute number of total nucleated cells (DAPI +) on each core at each time point.

### MPM whole samples staining and analysis

The whole section cohort of MPM samples were stained following the same conditions as the optimized mIF protocol. Similarly, we included a positive control (tonsil) and autofluorescence tissue (negative controls) during the staining of the cases.

**Figure 1:**
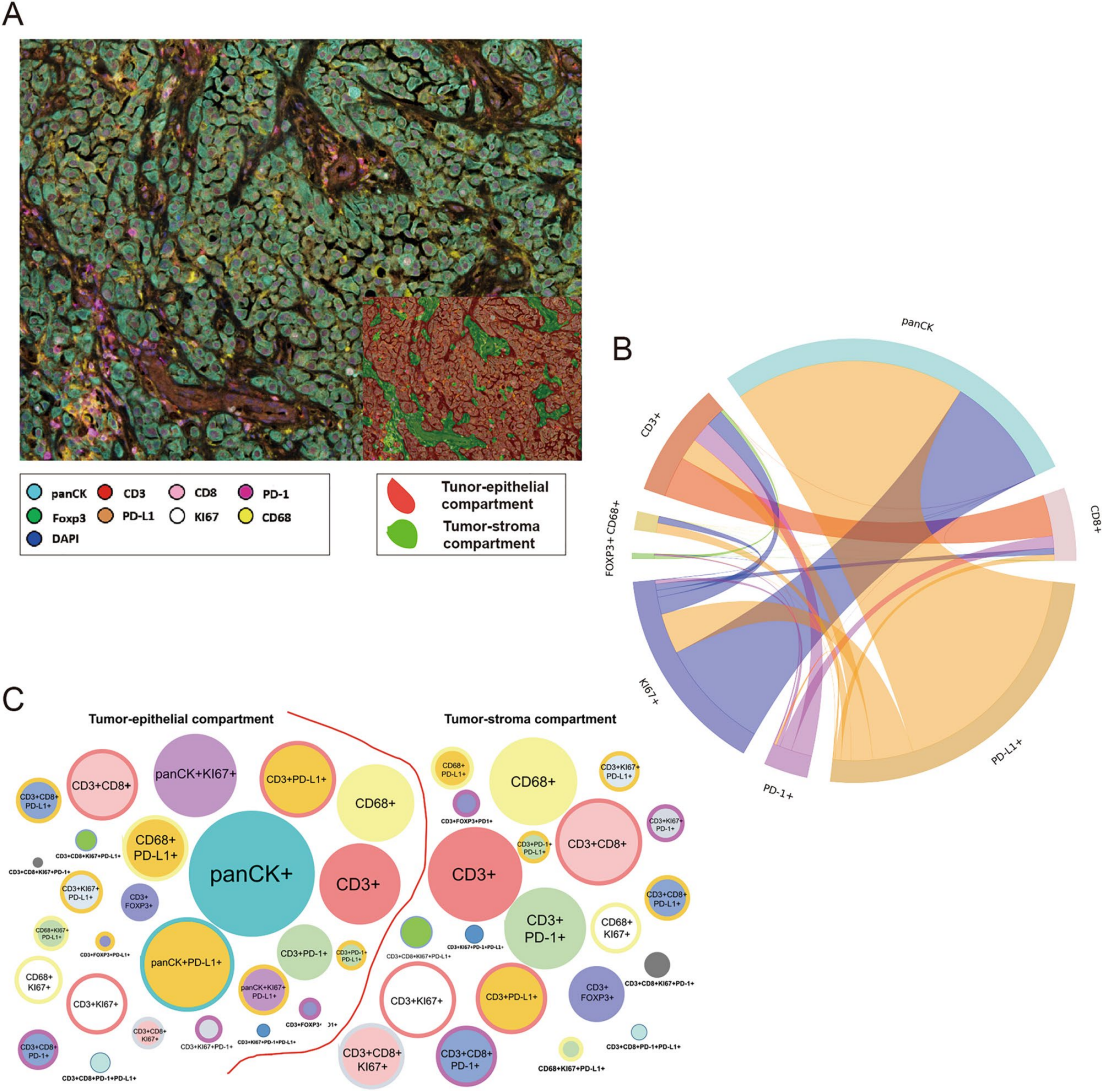
Representative microphotograph of multiplex immunofluorescence (mIF), graphic of markers’ co-expression and graphical representation comparing phenotypes between tumor-epithelial and tumor-stroma compartment in malignant pleural mesothelioma (MPM). (**A**) mIF image, inside, detail of compartmentalization of the tumor in tumor-epithelial (*red*) and tumor stroma (*green*) compartments. (**B**) Cord plot graphic showing the co-expression of the markers to generate the different cells phenotypes in the MPM cases. (**C**) Graphic representation of the relative densities of the different cell phenotypes in the tumor-epithelial and tumor-stroma compartments, as observed in the MPM cohort. Overall, the numbers of various immune cell phenotypes are higher in the tumor-stroma than in the tumor-epithelial compartment. mIF, 20 × magnification. The images were generated using Vectra/Polaris 3.0.3 scanner system, InForm 2.4.8 image analysis software (Akoya Biosciences), and R studio software version 3.6.1.

The slides were scanned using the Vectra/Polaris 3.0.3 (Akoya Biosciences) at low magnification, 10x (1.0 µm/pixel) through the full emission spectrum and using positive tonsil controls from the run staining to calibrate the spectral image scanner protocol. A pathologist selected 10 regions of interest (ROIs) for scanning in high magnification using the Phenochart Software image viewer 1.0.12 (931 × 698 µm size at resolution 20x) in order to capture various elements of tissue heterogeneity. Histologic assessment of each analysis area was performed for the tumor cases to ensure that the tumor tissue (at least 85% malignant cells, panCK +) was included in the selected intratumoral ROI. The different ROIs were divided according the expression of panCK or not in tumor-epithelial compartment (groups or nests of malignant cells) and tumor-stroma compartment (represented by the stroma area between tumor cells), respectively (Fig. 1A).

In the whole section mesothelioma cohort, the individual cells, defined by nuclei^24^ staining (DAPI +), were subjected to phenotyping to characterize co-localization of the multiple cell populations (Fig. 1B) detected using individual algorithms from the InForm 2.4.8 image analysis software under pathologist supervision. The individual image marker analyses from the panel were merged at the end using the X and Y coordinates of each cell by the program phenoptr script from R studio (Akoya Biosciences). Using SAS Enterprise Guide 7.1 software, the final report consolidation presented the average of the density per mm^2^ of the different cell phenotypes found from the ROIs analysed for the tumor epithelial and stroma compartments combined (tumor-epithelial-stroma compartment) and by compartment (tumor-epithelial and tumor-stroma compartments).

### Functional spatial distribution in MPM

To determine differences in cellular distribution within each compartment, the distance of each TAICs [T-cell phenotype CD3 + and macrophage phenotypes CD68 +) was calculated related to the malignant cells (panCK +)]. The matrix interaction from each case was created using X and Y coordinates from each cell phenotype using the R studio software version 3.6.1. The median distance from malignant cells to the different cells phenotypes observed was used to divide the different subpopulations to be geographical distributed as close to the malignant when their where located equal or less that the median radius and far when their where located more that the median radius. To evaluate cell interaction and spatial pattern distribution between malignant cells and different TAIC's, we compared the empirically derived nearest neighbor distance G function for marker point patterns to the theoretical Poisson function (median distances of the specific cells between cases) obtained by assuming the same intensity of the observed pattern. Furthermore, to characterize the probability to observe different patterns of cellular distribution across ROIs and patients we study the curves generate using the nearest neighbour distance G function and the theoretical Poisson curve.

### Statistical analysis

To verify the staining consistency each marker quantified from the TMA was correlated across all cores between the three time points using Spearman’s rank correlation coefficients. The *P*-values obtained for each marker on each core between three time points were adjusted by Bonferroni correction, and significance was called when adjusted *P*-value < 0.05.For the whole sections, the chi-square test or Fisher exact test was used to examine differences in categorical variables, and the Wilcoxon rank-sum test and Kruskal–Wallis test were used to explore differences in continuous variables between groups of patients with MPM. To determinate the interaction between cells phenotypes and to characterize the patterns of distribution according the nearest neighbour distance G function and the theoretical Poisson curve we applied the Hidden Markov algorithm to observe the fuzzy intersection of the different curves generated by the distribution of different cell phenotypes. The statistical software programs R studio (version 3.6.1) and IBM SPSS (version 22; Armonk, NY) were used to perform the computations for all analyses.

### Ethics approval and consent to participate

The study was approved by the MD Anderson Institutional Review Board, and consent for participation and publication was obtained from all the patients included in the study and is available for review at any time.

## Results

### Optimization of Immune markers in the mIF panel

Using chromogenic IHC and single IF approaches, we evaluated the different markers to determine whether similar patterns of staining are obtained with both immunohistochemical techniques. Consistent with previous reports, PD-L1 showed membranous expression in epithelial tonsil crypts^26^, as shown in the microphotographs in Supplementary Fig. 1. Likewise, the other markers showed similar staining patterns with mIF compared with IHC stains in the tonsil controls—epithelial cell marker panCK was expressed by epithelial cells, the pan T-cell marker CD3 was the most abundant among cells surrounding the germinal centers followed by CD8, and Foxp3 (nuclear expression). PD-1 was observed mostly distributed in the germinal centers, as well as the proliferation marker KI67 (nuclear expression) was conglomerate in this location and diffusely surrounding that. Finally, macrophage marker CD68 showed predominant expression also in the germinal centers of the control tonsils, Supplementary Fig. 1.

### Staining consistency

To observe the staining consistency of the mIF panel we stained the panel three times using a TMA with MPM cases, in consecutive weeks and then we compared the quantification of the individual markers form each core across the time (Supplementary Fig. 2).

Although, as expected geographic change distributions of cells, expressing those markers, was observed during the quantification, the mIF staining across the time exhibited positive and significant correlations overall between week 1 *vs* week 2, week 1 *vs* week 3 or week 2 *vs* week 3 (Supplementary Table 3). Indeed, the individual markers showed high consistency and reproducibility across the time and cores (Supplementary Fig. 3).

### Patients’ clinicopathologic characteristics

Supplementary Table 1 shows the clinicopathologic characteristics of our exploratory cohort of MPM. According to the tumor morphology, 11 cases were characterized as epithelioid mesotheliomas and one as biphasic mesothelioma. All the patients received platinum and pemetrexed neoadjuvant chemotherapy followed by surgical resection with the intent of macroscopic cytoreduction. Adjuvant chemotherapy and radiotherapy also were given in 6 and 5 patients, respectively.

### Immune cell phenotypes characterized in MPM using whole section samples

**Figure 2:**
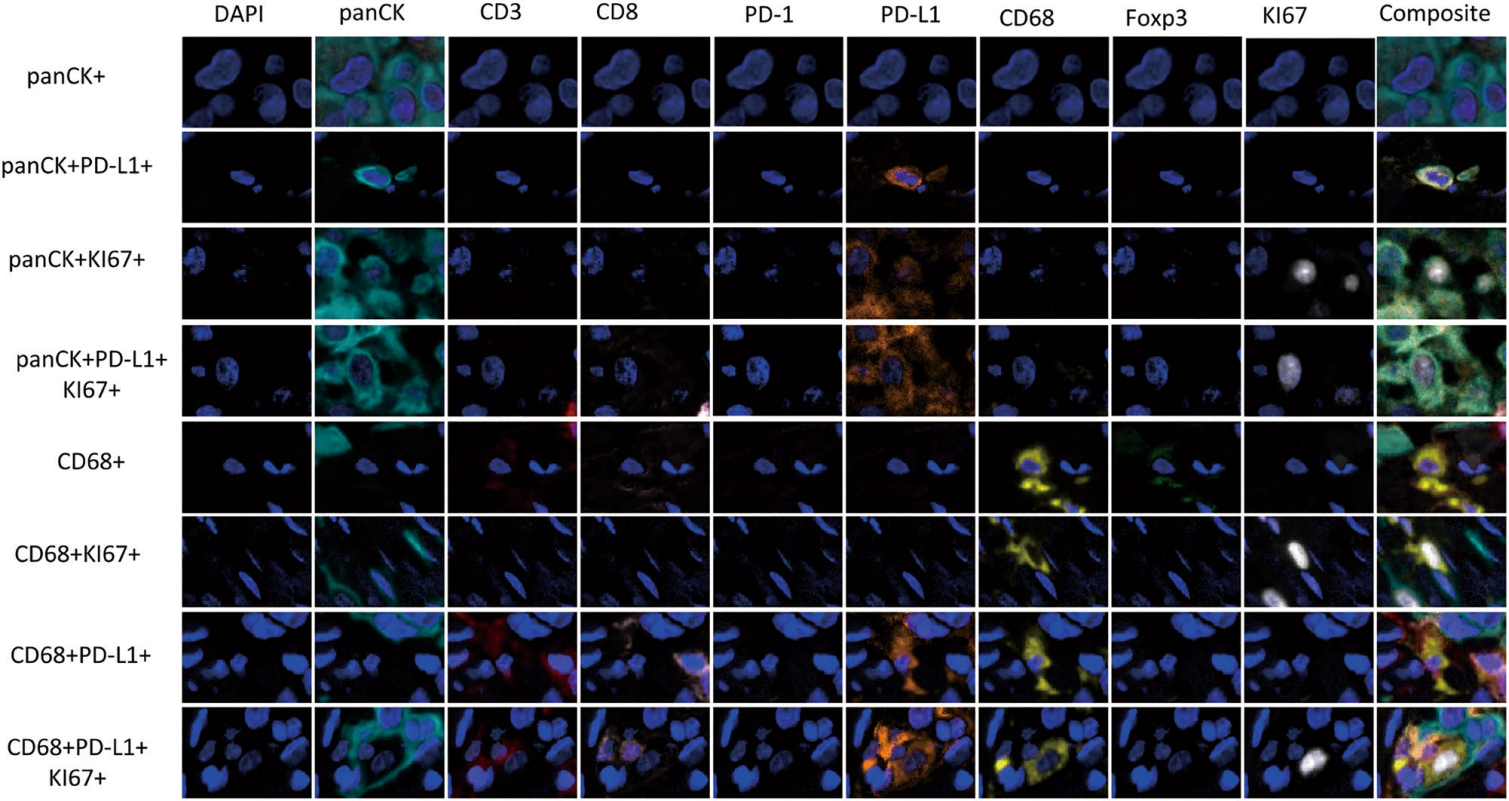
Microphotographs of representative examples of co-localization of malignant cells (panCK +) and macrophages (CD68 +) cells populations observed with the multiplex immunofluorescence panel in the malignant pleural mesothelioma cohort. The images were generated using Vectra/Polaris 3.0.3 scanner system and InForm 2.4.8 image analysis software (Akoya Biosciences).

**Figure 3:**
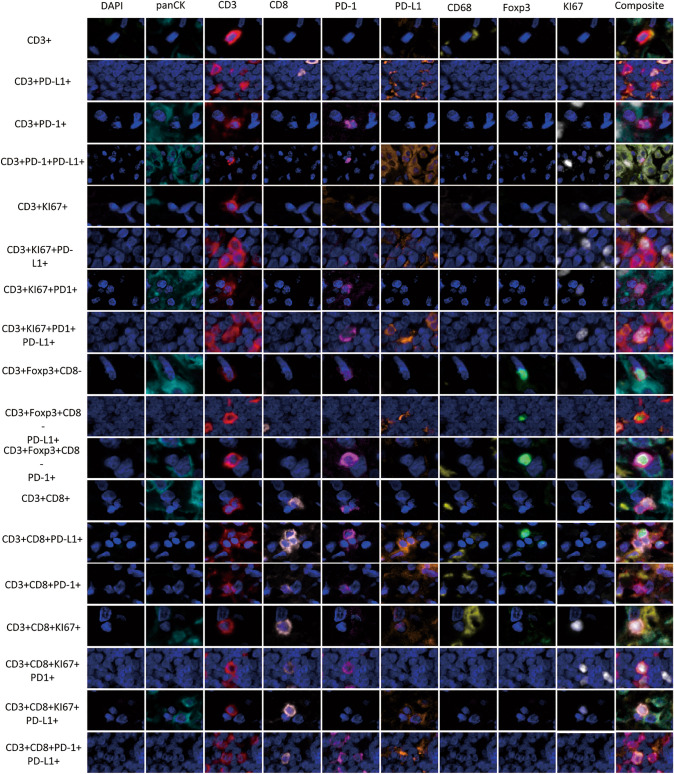
Microphotographs of representative examples of co-localization. Different CD3 + T-cell subpopulations observed with the multiplex immunofluorescence panel in the malignant pleural mesothelioma cohort. The images were generated using Vectra/Polaris 3.0.3 scanner system and InForm 2.4.8 image analysis software (Akoya Biosciences).

In studying the TME from MPM, it was possible to identify different TAIC populations using the expression of cell-type specific markers of CD3, CD68, and panCK and their co-expression with the other markers in the panel, as shown in Figs. 2 and 3. In this cohort, all cases were classified as PD-L1 +, with a cutoff of greater than 1% of the malignant cells expressing PD-L1. Interestingly, we observed that an only a median of 143.00 cell/mm^2^ [minimum (min) 0 cell/mm^2^; maximum (max) 524.14 cell/mm^2^] of the total panCK^+^ cells co-expressed KI67, and of those, only a median of 9.03 cell/mm^2^ (min 0 cell/mm^2^; max 318.89 cell/mm^2^) expressed PD-L1 + (panCK + KI67 + PD-L1 +), showing active proliferation of a small number of tumor cells, Table 1.

**Table 1:** Different cell phenotypes densities according tumor compartments (epithelial, stoma and epithelial-stroma compartment) in malignant pleural mesothelioma patients (N = 12).

Phenotype	Median cell densities by tumor compartment (cells/mm^2^)			
	Epithelial	Stroma	P*	Epithelial-Stroma
panCK +	2312.10	0.00	–	1471.03
panCK + PD-L1 +	661.54	0.00	–	421.22
panCK + KI67 +	294.19	0.00	–	143.00
panCK + KI67 + PD-L1 +	13.24	0.00	–	9.03
CD3 +	292.94	511.67	0.014	428.19
CD3 + PD-L1 +	45.12	11.42	0.003	38.55
CD3 + PD-1 +	15.34	32.94	0.114	26.69
CD3 + PD-1 + PD-L1 +	2.52	0.93	0.178	2.41
CD3 + KI67 +	17.22	30.75	0.143	19.05
CD3 + KI67 + PD-L1 +	6.45	3.37	0.551	5.17
CD3 + KI67 + PD-1 +	1.31	2.87	0.128	2.08
CD3 + KI67 + PD-1 + PD-L1 +	0.61	0.54	0.799	0.75
CD3 + Foxp3 + CD8 −	3.88	7.09	0.114	4.91
CD3 + Foxp3 + CD8 − PD-L1 +	0.72	0.00	< 0.001	0.39
CD3 + Foxp3 + CD8 − PD-1 +	0.88	0.65	1.000	1.00
CD3 + CD8 +	32.57	97.18	0.002	57.67
CD3 + CD8 + PD-L1 +	9.98	5.35	0.128	7.67
CD3 + CD8 + PD-1 +	4.45	7.97	0.219	7.80
CD3 + CD8 + PD-1 + PD-L1 +	1.12	0.26	0.321	1.13
CD3 + CD8 + KI67 +	2.92	9.47	0.045	5.89
CD3 + CD8 + KI67 + PD-L1 +	1.29	1.77	0.443	1.74
CD3 + CD8 + KI67 + PD-1 +	0.38	0.78	0.266	0.57
CD68 +	176.75	110.96	0.843	143.89
CD68 + KI67 +	13.10	6.91	0.347	9.75
CD68 + KI67 + PD-L1 +	3.58	0.57	0.114	2.54
CD68 + PD-L1 +	17.63	4.08	0.045	15.68

*P, comparison between tumor-epithelial and tumor-stroma compartment.

The dominant CD3 + T-cell subset observed in this MPM cohort (tumor-epithelial and tumor-stroma compartments) were cytotoxic T cells (CD3 + CD8 +; median, 57.67 cell/mm^2^; min 26.23 cell/mm^2^; max 175.35 cell/mm^2^). We also observed regulatory T cells (CD3 + Foxp3 + CD8 −, median 4.91 cell/mm^2^, min 0 cell/mm^2^, max 524.14 cell/mm^2^), antigen-experienced T cells (CD3 + PD-1 +, median 26.69 cell/mm^2^, min 5.08 cell/mm^2^, max 193.99 cell/mm^2^) and PD-L1 + T cells (CD3 + PD-L1 +, median 38.55 cell/mm^2^, min 5.66 cell/mm^2^, max 89.26 cell/mm^2^), demonstrating potential T-cell mediated suppressive axes within the MPM TME. Other phenotypes observed were antigen-experienced cytotoxic T cells (CD3 + CD8 + PD-1 +), and PD-L1 + cytotoxic T cells (CD3 + CD8 + PD-L1 +). Additionally, CD68 + macrophages expressing PD-L1 were present with a subset showing co-expression of KI67 (CD68 + KI67 + PD-L1 +, Table 1). As expected, the densities of TAICs were higher overall in the tumor-stroma compartment than in the tumor-epithelial compartment as shown in Fig. 1C and Table 1. Interestingly, in the tumor-epithelial compartment, the densities of cytotoxic T cells (CD3 + CD8 +) were higher when compared with the tumor-stroma compartment. In addition, proliferating, cytotoxic T-cells (CD3 + CD8 + KI67 +), PD-L1 + T-cells (CD3 + PD-L1 +) and macrophages (CD68 +) expressing PD-L1 were more prevalent in the tumor-epithelial compartment than in the tumor-stroma compartment.

Although, the small number of MPM cases included for the exploratory study, we observed that tumors obtained from ever-smokers had significantly higher densities of T-cells (CD3 + CD8 + KI67 +, CD3 + CD8 + PD-L1 +, and CD3 + Foxp3 + CD8 − PD-L1 +) than never-smoker patients (*P* = 0.048, *P* = 0.018, and *P* = 0.018, respectively). No other correlations were observed.

### Exploratory functional spatial distribution in MPM tumors

**Figure 4:**
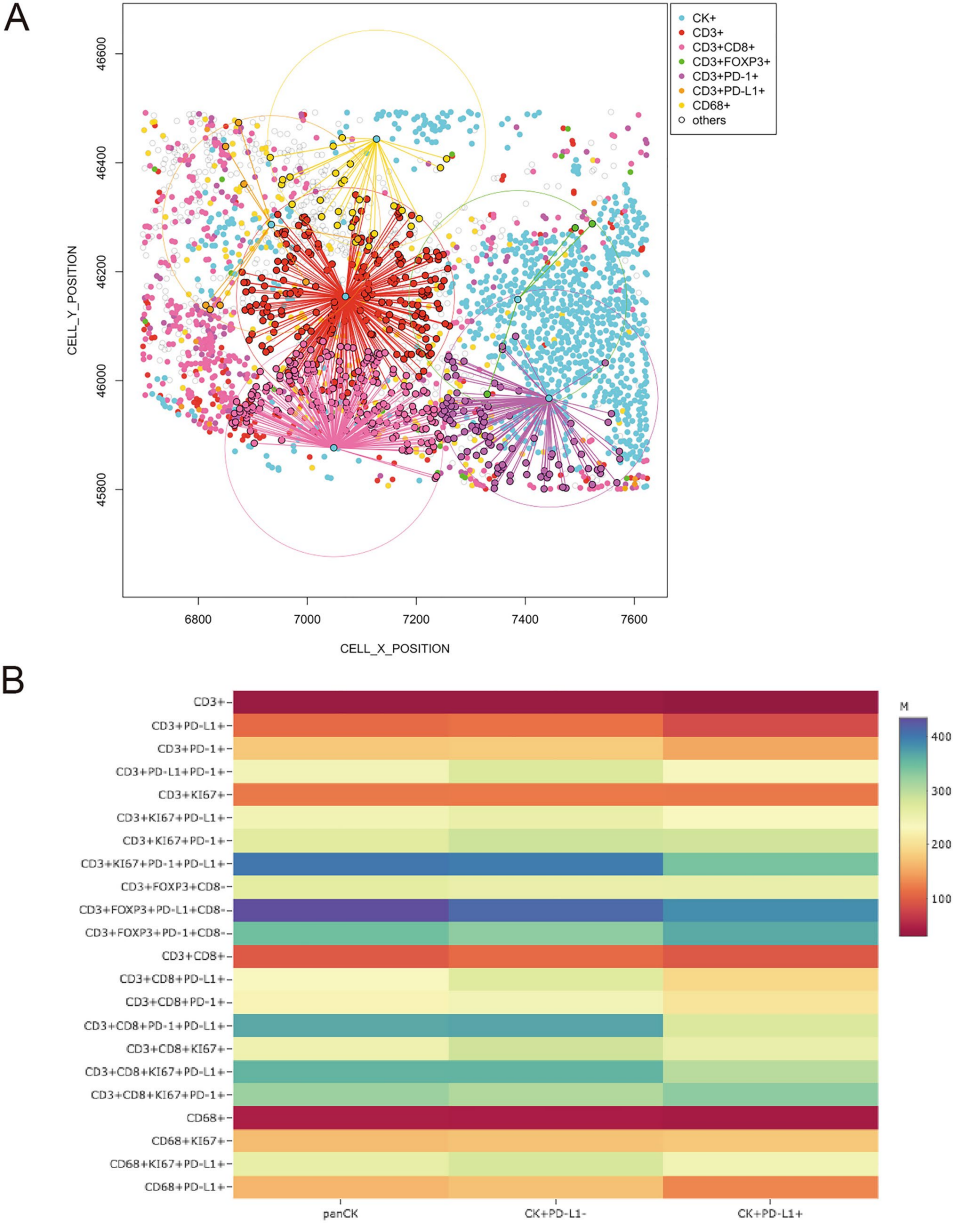
Spatial analysis showing, (**A**) e representative examples of distance measurements from malignant cells (panCK +) to different T-cells CD3 + phenotypes using the X and Y coordinate; (**B**) head map showing the relative proximity from panCK + cells, *first column*; panCK + cells that not expressing PD-L1, *second column*; and panCK + cells that expressing PD-L1, *third column,* to a variety tumor associated immune cell phenotypes in the malignant pleural mesothelioma cases. The images were generated using R studio software version 3.6.1.

To map the spatial organization of the TAICs, we assessed their distribution using the X and Y coordinates of each cell within the tumor-epithelial or tumor-stromal compartment (Fig. 4A). In order to capture the interaction between cells, we constructed a matrix where each entry is the euclidean distance from a pair of cells. From this matrix, we were able to identify the median distance from malignant cells (panCK +) to the multiple TAIC phenotypes described above. Using the median distance, we were able to generate a heat map including the different TAICs obtained with the image analysis and we identified the distance of 243.46 microns as overall radius distance from panCK + and the TAICs. With that, we are able to consider all the TAICs inside of that radius as a closed TAICs from malignant cells and the TAICs outside that radius as far to the malignant cells. In our cohort of MPM tumors, the closest TAIC phenotypes to panCK + malignant cells were the cytotoxic T-cells (CD3 + CD8 +) with a median distance of 94.28 microns. We also observed that T-cells expressing PD-L1 (CD3 + PD-L1 +), antigen experienced T-cells (CD3 + PD-1 +), and macrophages expressing PD-L1 were relatively close to malignant cells (median distance, 110.32, 176.46, and 161.88 microns, respectively). Regulatory T-cells (CD3 + Foxp3 + CD8-, median distance, 265.45 microns) and cytotoxic T-cells antigen-experienced (CD3 + CD8 + PD-1 +, median distance, 386.90 microns) were detected as far TAICs from malignant cells (Fig. 4B *first column* and Table 2). Furthermore, when we compared the overall median distance of TAICs from PD-L1 + (234.00 microns) versus PD-L1^-^ (264.51 microns) malignant cells, we observed that cytotoxic T-cells (CD3 + CD8 +) and antigen experienced T-cells (CD3 + PD-1 +) were closer in proximity to PD-L1 + malignant cells as compared to those lacking PD-L1 expression, Fig. 4B *second* and *third column* and Table 2).

**Table 2:** Median distance in micros from malignant cell (panCK) phenotypes to tumor associate immune cell (TAIC) phenotypes observed in the malignant pleural mesothelioma cases.

To TAICs phenotypes	From panCK phenotypes		
	panCK +	panCK + PD-L1-	panCK + PD-L1 +
	Median distance in micros		
CD3 +	33.75	33.46	30.74
CD3 + PD-L1 +	110.33	115.52	84.12
CD3 + PD-1 +	176.42	179.12	151.83
CD3 + PD-L1 + PD-1 +	242.47	274.28	234.86
CD3 + KI67 +	120.99	120.69	121.16
CD3 + KI67 + PD-L1 +	244.45	258.04	233.15
CD3 + KI67 + PD-1 +	267.98	287.58	284.34
CD3 + KI67 + PD-1 + PD-L1 +	402.88	397.64	342.28
CD3 + Foxp3 + CD8-	265.45	259.67	258.69
CD3 + Foxp3 + PD-L1 + CD8-	435.18	413.67	385.55
CD3 + Foxp3 + PD-1 + CD8-	347.43	329.64	365.78
CD3 + CD8 +	98.29	109.78	96.00
CD3 + CD8 + PD-L1 +	232.38	269.36	191.24
CD3 + CD8 + PD-1 +	224.94	242.95	206.57
CD3 + CD8 + PD-1 + PD-L1 +	367.26	369.87	275.59
CD3 + CD8 + KI67 +	249.46	285.03	258.71
CD3 + CD8 + KI67 + PD-L1 +	356.88	357.77	300.75
CD3 + CD8 + KI67 + PD-1 +	321.10	306.60	330.10
CD68 +	47.27	46.75	42.00
CD68 + KI67 +	167.88	172.60	176.39
CD68 + KI67 + PD-L1 +	261.41	278.44	245.89
CD68 + PD-L1 +	161.88	171.60	129.52

### Identification of distinct spatial TAIC patterns

**Figure 5:**
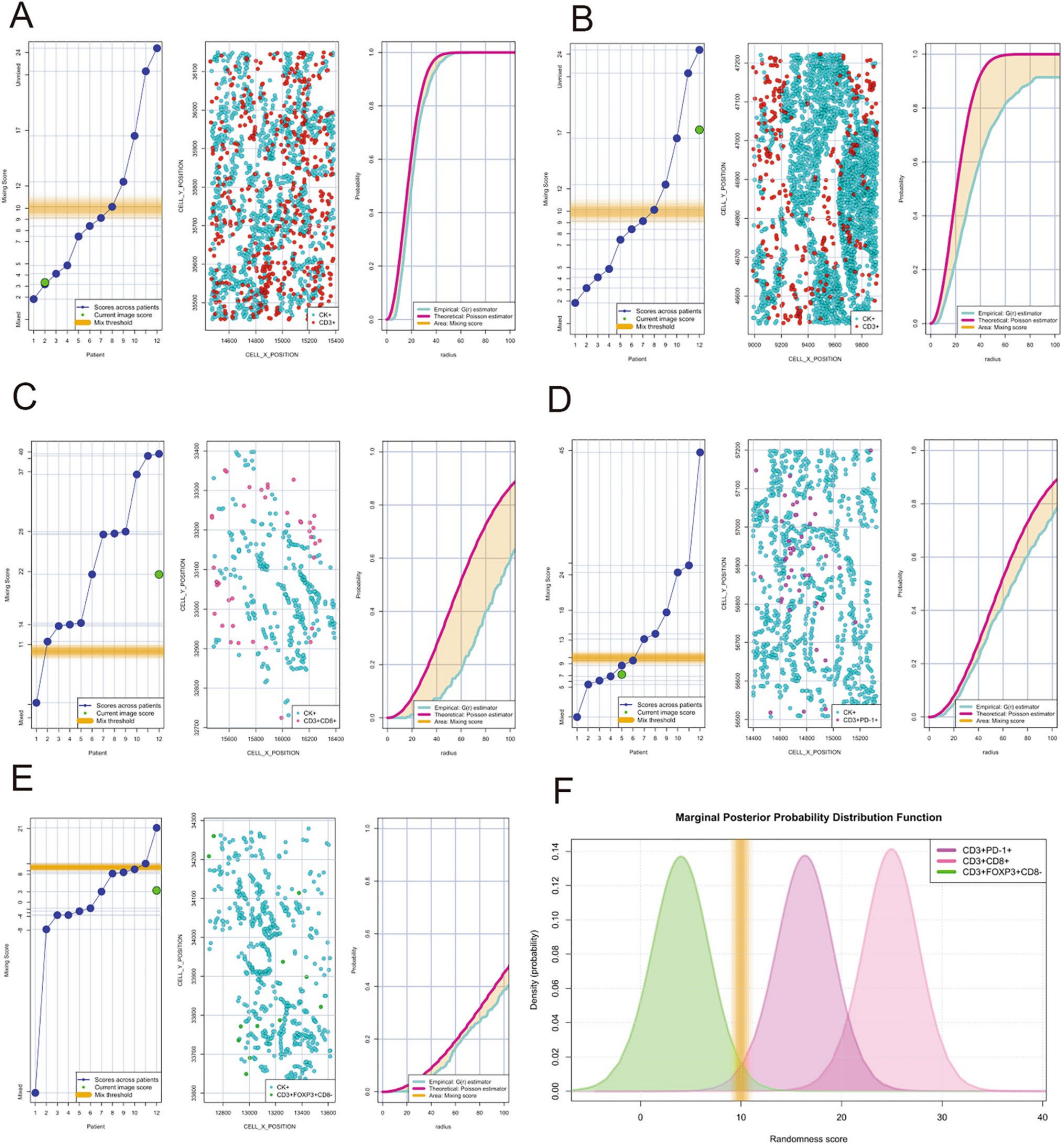
Individual nearest neighbor distance G function and theoretical Poisson curves graphics for the identification of different cellular patterns of distance distribution from malignant cells (panCK +) to T-cells CD3 + phenotypes. (**A** to **E**) showing the scoring scale that the image represent across the cases and the threshold to be considered mixed or unmixed pattern (*left side*) , graphic distribution representation of the T-cell phenotypes and panCK cells (*middle side*) and the nearest neighbor distance G function and theoretical Poisson curves area (*right side*). (**A**) Mixed pattern distribution between T-cells CD3 + and panCK + cells, (**B**) unmixed pattern distribution between T-cells CD3 + and panCK + cells, (**C**) unmixed pattern distribution between cytotoxic T-cells (CD3 + CD8 +) and panCK + cells, (**D**) mixed pattern distribution between antigen experienced T-cells (CD3 + PD + 1) and panCK + cells, (**E**) mixed pattern distribution between regulatory T-cells (CD3 + Foxp3 + CD8-) and panCK + cells, and (**F**) graphical model showing the spatial distribution interaction between cytotoxic T-cells, antigen experienced T-cells and regulatory T-cells related to malignant cells showing the different curves of distribution according the T-cell phenotypes analysed. The images were generated using R studio software version 3.6.1.

To determine whether distinct TAIC patterns may be identified in the TME using this novel mIF panel, we analysed the nearest neighbour distance in increasing radiuses from malignant cells (30, 50, 75, 100 and 200 microns). This analysis showed that the number of TAIC phenotypes gradually increases as they are further from malignant cells. Combining the empirically derived nearest neighbour distance G function curve from the CD3 + T-cells and panCK + malignant cells to the theoretical Poisson function curve, we identified two patterns of cellular distribution, mixed and unmixed. A mixed pattern was defined as when the curve generated by the interaction of the CD3 + T-cells and panCK + malignant cells ranges from -10 to + 10 relative to the Poisson curve. An unmixed pattern was defined as when the curve generated with the CD3 + T-cells and panCK + malignant cell interaction was greater than + 10 relative to the Poisson curve. The mixed pattern was characterized by the close interaction between malignant cells and T-cells with a homogenous distribution of distances between those cells. This pattern was observed in 7 cases out of 12 cases (Fig. 5A, score, *left*; cell distribution, *middle*; curves, *right side*). In contrast, the unmixed pattern showed low interaction between malignant cells and T cells. This pattern was defined by cohesive nests of malignant cells with very low T-cell interaction (n = 5 out 12 cases, Fig. 5B, score, *left*; cell distribution, *middle*; curves, *right side*). When we assessed the distribution pattern of specific key TAIC phenotypes defined in this panel, the cytotoxic T-cells (CD3 + CD8 +) overall showed an unmixed pattern suggesting a poor or weak interaction with malignant cells, (Fig. 5C, score, *left*; cell distribution, *middle*; curves, *right side*). Conversely, although antigen experienced T cells (CD3 + PD-1 +, Fig. 5D, score, *left*; cell distribution, *middle*; curves, *right side*) and regulatory T cells (CD3 + Foxp3 + CD8-, Fig. 5E, score, *left*; cell distribution, *middle*; curves, *right side*) were found at a lower frequency than their cytotoxic T-cell counterparts, these cell types showed a mixed pattern. When we studied the distribution of these curves from those specific phenotypes across ROIs and cases, we observed that those patterns of cellular distribution were frequently repeated; thus, characterizing a specific landscape in the MPM cohort showing that antigen experienced T cells and regulatory T cells have an opposite spatial distribution when compared to their cytotoxic T-cell counterparts as related to their interaction with malignant cells Overall this suggests that cytotoxic T cells have a low probability of interaction with malignant cells and antigen experienced T cells and regulatory T cells showed a have high probability of interaction with malignant cells (Fig. 5F).

## Discussion

In the last 5 years mIF has been shown to be an invaluable tool for tumor tissue immune profiling to identify multiple biological markers on a single tissue. Using control tissues as well as systematic antibody optimization by IHC, single IF, and then mIF^25^, we developed an automated nine-colour mIF panel to visualize and characterize deeper the TME in paraffin samples. Additionally, as we previously showed^25^, proper balance of the different fluorophores linked with a specific antibody can avoid cross-talking reaction and generate a clear signal between markers to obtained a consistence staining correlation across the time. In the whole section cohort of MPMs, we were able to assess, with extraordinary fidelity according to the antibodies included in the panel, several TAIC phenotypes, showing that we successfully multiplexed these biomarkers by following our protocol. These results demonstrate the practical scalability of this method for studying different aspects of the TME in paraffin tissues, and the data generated with the image analysis from the tissue can be used to explore the spatial distribution of the cells using mathematical models to generate more comprehensive characterization of the TME as described below.

Following the steps described for automated staining, we obtained reproducible results across the different weeks. The methodology described can be performed in 1 day, 17 h. for those eighth Opal fluorophore tyramides linked with their antibody plus DAPI. Overall, we observed highly reproducibility between markers with correlation sometimes more than 0.90 Rho across de different time points using the protocol. Although, as expected, geographic variability of the cells expressing those markers were observed specially with marker with low expression, as Foxp3, we showed a good and significant reproducibility across the time of staining. Furthermore, this protocol can be handled easily, avoiding fluctuations in the level of specific signals from the different marker used, or from the background at the end of the optimization process.In the exploratory side using this protocol and whole sections sample for the analysis, we observed low TAIC densities in our cohort of MPMs compared with our previous studies with other lung tumor types^27, 28^. However, although the number of cases used in this study was small, we were able to identify specific features of TAIC subsets interacting with malignant cells. In fact, while higher densities of cytotoxic T cells were observed relative to the others TAIC subsets, this subset was not found to interact strongly with malignant cells. T regulatory cells as well as antigen-experienced, PD-1 + /PD-L1 + T cells were more likely to interact with malignant cells.

The PD-1/PD-L1 axis was also found to guide specific cellular interactions. In fact, we observed that malignant cells expressing PD-L1 were spatially closely associated with CD3 + CD8 + cytotoxic T cells and total CD3 + PD-1 + T cells as compared to their PD-L1- counterparts. In addition, while CD68 + macrophages were found to closely associate with tumor cells, PD-L1 + macrophages were found to have the closest interaction with PD-L1 + tumor cells. Although previous studies showed that PD-L1 expression by MPM oscillates between 16 and 40% according to histologic subtype, in our small cohort, all cases showed PD-L1 + expression by malignant cells at a cutoff of more than 1%^29–31^, we hypothesize that this increase in PD-L1 may be related to the neoadjuvant chemotherapy as shown previously in lung cancer^32^ and MPMs^33^. Overall, neoadjuvant chemotherapy tends to increase the expression of PD-L1 in malignant cells and TAICs. Interestingly, we observed that ever-smoker patients showed overall higher densities of active proliferation of cytotoxic T-cells (CD3 + CD8 + KI67 +) and T-cell phenotypes expressing PD-L1, suggesting that smoking status affects the adaptive immunity and can also increase the PD-L1 expression in MPM as shown by Patil and colleagues^34^.

Although, cytotoxic T cells were the subpopulation of TAICs observed closest to tumor cells in our MPM cohort, other TAICs like antigen experienced T cells (CD3 + PD-L1 +, CD3 + PD-1 +), regulatory T cells (CD3 + Foxp3 + CD8−), cytotoxic T cells antigen experienced (CD3 + CD8 + PD-1 +) and macrophages (CD68 +) expressing PD-L1 were also observed in close proximity from the malignant cells. It is well established that certain tumors and the surrounding cellular composition can generate an immunosuppressive microenvironment to suppress the effector function of cytotoxic T-cells inside the tumor as well as inhibit the ability of antigen-presenting cells to induce anti-tumor T-cell responses^35–37^. Immunosuppressive factors that contribute to tumor infiltrating T cell dysfunction in the TME include presence and frequency of regulatory T cells, presence of immature antigen presenting cells, expression of inhibitory receptors and their ligands, and production of immunosuppresive cytokines^38, 39^. All these factors in our mesothelioma cohort might have contributed to the localization patterns observed in these cases. Interestingly, we also observed that malignant cells expressing PD-L1 are closely localized with cytotoxic T-cells (CD3 + CD8 +) and antigen experienced T-cells (CD3 + PD-1 +) while these subsets are less likely to interact with PD-L1 negative malignant cells. This suggests an active engagement of this suppressive axis that may be inhibiting the anti-tumor immune response.

The spatial analysis per radius also showed overall the small quantity of TAICs in the intratumoral compartment. According to the geographic distribution of the T cells and tumor cells, two cellular patterns were identified, a mixed pattern characterized by high interaction between malignant cells and T-cells and an unmixed pattern characterize by low interaction between malignant cells and T cells. Finally, similar to that observed by Ahmadzadeh and colleagues^40^, our spatial distribution analysis demonstrated that although cytotoxic T-cells (CD3 + CD8 +) could be found close to malignant cells, this was in a unmixed pattern suggesting a reduced interaction. In contrast, PD-1 + T cells and regulatory T cells were present in a mixed pattern suggesting an active interaction with malignant cells.

## Conclusion

In summary, we successful showed the reproducibility of this methodology and their incorporation for immune profiling TME into translational studies to refine our ability to understand the cellular behavior within solid tumors and predict new treatment strategies. The analysis of immune infiltrates by mIF serves as a quantitative, automated tool, allowing multiplexed analysis of density and spatial distribution of several markers simultaneously to identify specific immune cell types in different tumor compartments and it could provide important knowledge for translational pathology studies. Although we showed the potential of this technique in small cohort of MPM, future validation is necessary using a similar cohort with a large set of patients to corroborate the correlations observed in this tumor tissue type. The main limitation for our exploratory analysis in our study is the small number of MPM cases used but it was minimized by the data obtained using this revolutionary technique and the image analysis applied. The present study is largely descriptive and exploratory, and extension of our findings are essential.

## Supplementary Information

Supplementary Information.

Supplementary Fig. 1.

Supplementary Fig. 2.

Supplementary Fig. 3.

Supplementary Fig. 4.

Supplementary Table 1.

Supplementary Table 2.

Supplementary Table 3.

## Data Availability

The raw datasets used and/or analyzed during the current study are available from the corresponding author on reasonable request.

## Abbreviations

FFPE: Formalin-fixed, paraffin-embedded
IHC: Immunohistochemistry
mIF: Multiplex immunofluorescence
MPM: Malignant pleural mesothelioma
PD-1: Programmed cell death protein 1
PD-L1: Programmed cell death ligand 1
TAIC: Tumor-associated immune cell
TME: Tumor microenvironment
