# Supplementary material for: Identification of distinct immune landscapes using an automated nine-color multiplex immunofluorescence staining panel and image analysis in paraffin tumor tissues
Source: Sci Rep. 2021 Feb 25;11:4530. doi: 10.1038/s41598-021-83858-x (PMC7907283; doi:10.1038/s41598-021-83858-x)
Supplement: Supplementary file 1 — Supplementary Information. [file 41598_2021_83858_MOESM1_ESM.docx]

**Supplementary Figure 1.** Microphotographs of representative examples of individual IHC and multiplex immunofluorescence (mIF) markers in tonsil tissue. Showing an equal pattern of distribution observed with each individual marker between IHC and the mIF panel in a tonsil tissue. 20x magnification. The images were generated using Vectra/Polaris 3.0.3 scanner system and InForm 2.4.8 image analysis software (Akoya Biosciences).

**Supplementary Figure 2.** Microphotographs of the spectral library showing the fluorescence extraction from the individual fluorophores used in the panel. The images were generated using Vectra/Polaris 3.0.3 scanner system and InForm 2.4.8 image analysis software (Akoya Biosciences).

**Supplementary Figure 3.** Microphotographs of representative example of multiplex immunofluorescence (mIF) markers from a core of the TMA across the three time points of staining (Week 1, Week 2 and Week 3). Inside detail showing the cellular staining pattern of each individual marker as well as in the composite image that include all the markers together. Malignant pleural mesothelioma tissue, mIF 20x magnification. The images were generated using Vectra/Polaris 3.0.3 scanner system and InForm 2.4.8 image analysis software (Akoya Biosciences).

**Supplementary Figure 4.** Trellis plots showing the staining consistency across the three time points from each individual marker, core by core and between weeks from the TMA of malignant pleural mesothelioma cases. The blue diamond represents week 1, the red square week 2 and the green triangle represents week 3. The images were generated using R studio software version 3.6.1.
