# Supplementary figures and images for: Identification of distinct immune landscapes using an automated nine-color multiplex immunofluorescence staining panel and image analysis in paraffin tumor tissues
Source: Sci Rep. 2021 Feb 25;11:4530. doi: 10.1038/s41598-021-83858-x (PMC7907283; doi:10.1038/s41598-021-83858-x)

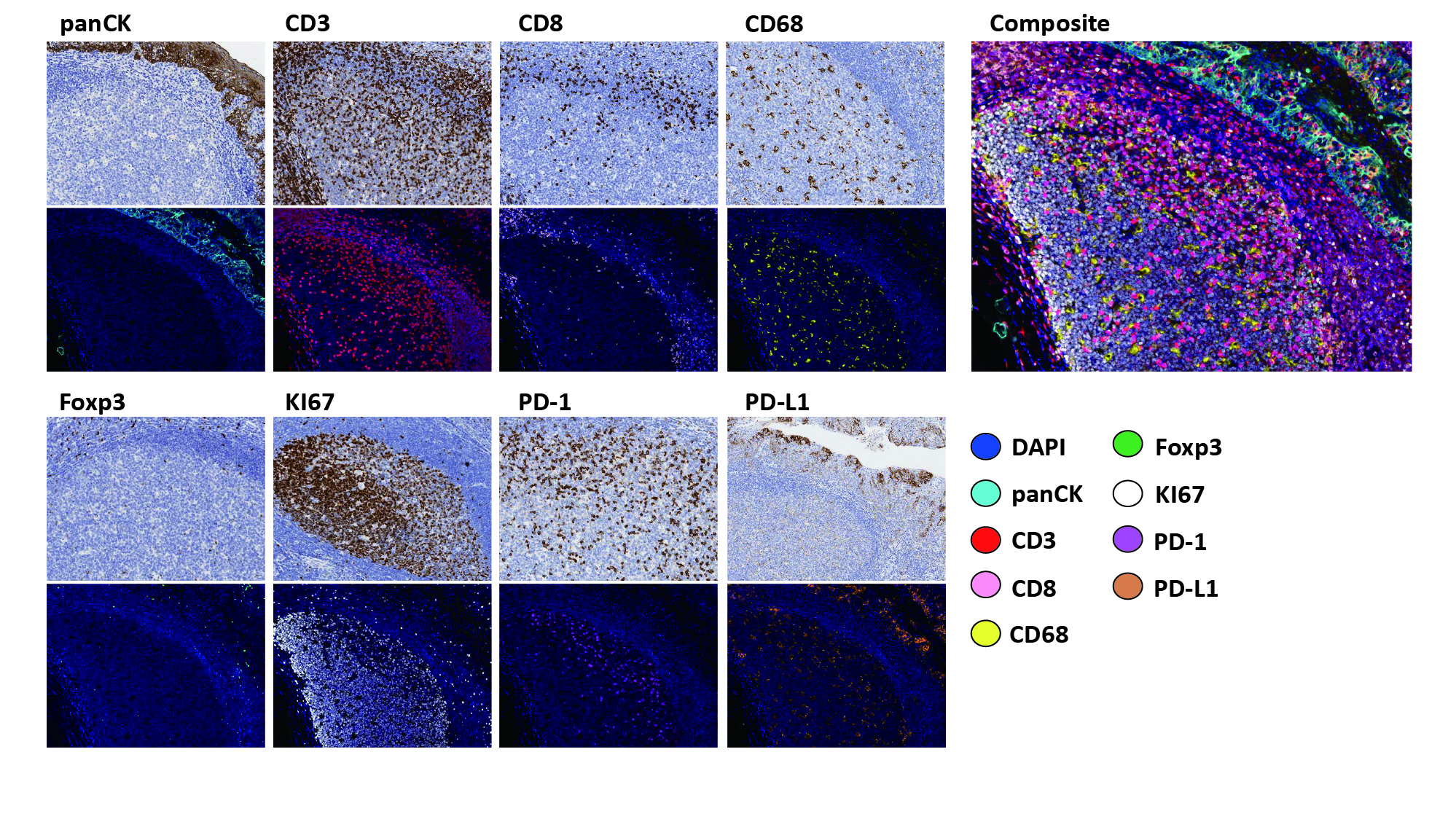

Supplement: Supplementary file 2 — Supplementary Fig. 1. [file 41598_2021_83858_MOESM2_ESM.tif]

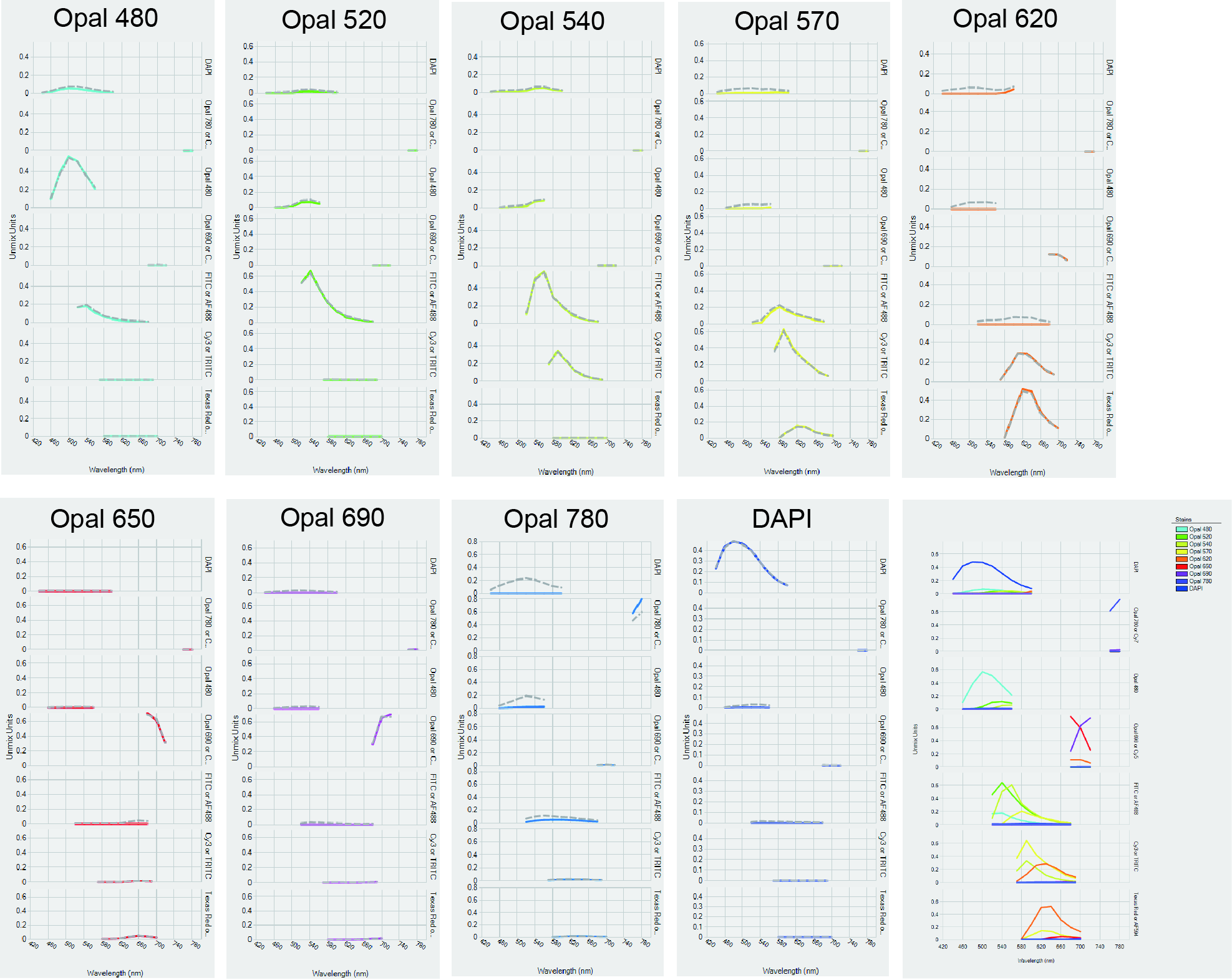

Supplement: Supplementary file 3 — Supplementary Fig. 2. [file 41598_2021_83858_MOESM3_ESM.tif]

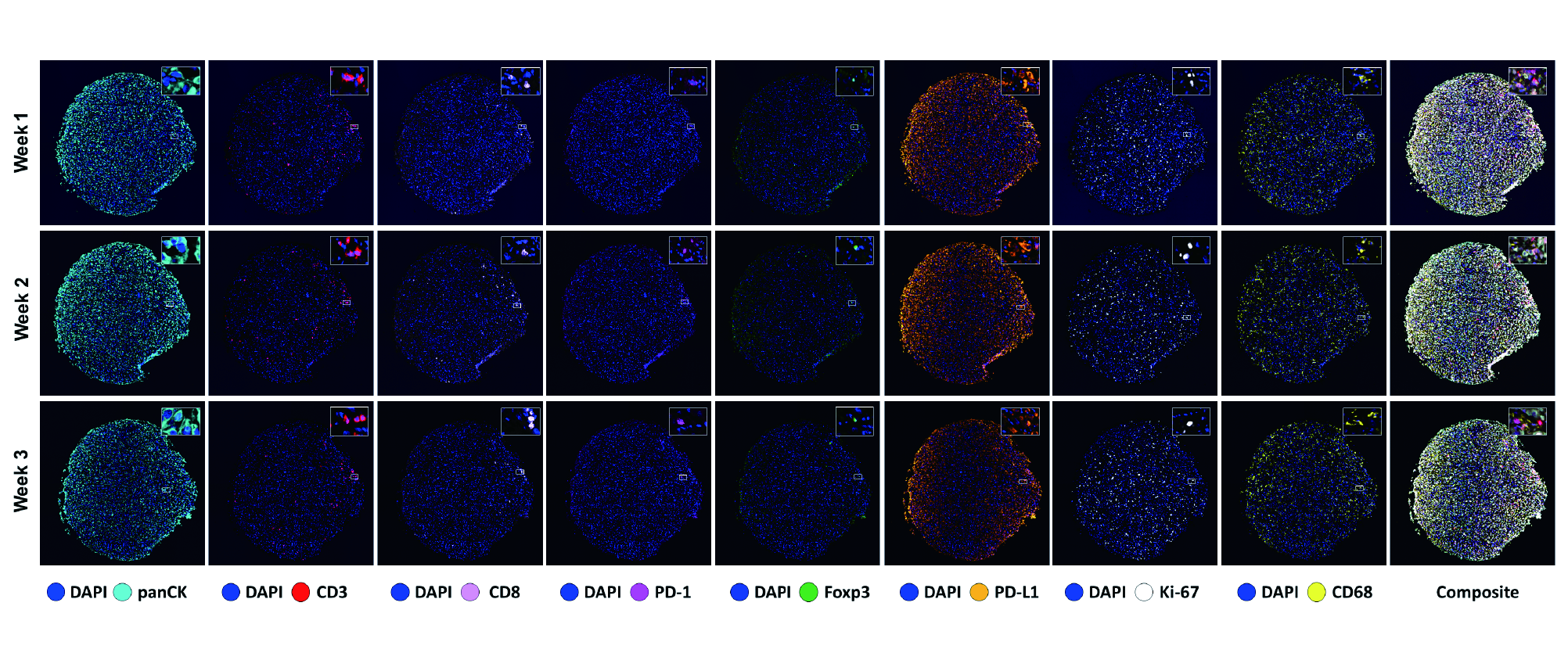

Supplement: Supplementary file 4 — Supplementary Fig. 3. [file 41598_2021_83858_MOESM4_ESM.tif]

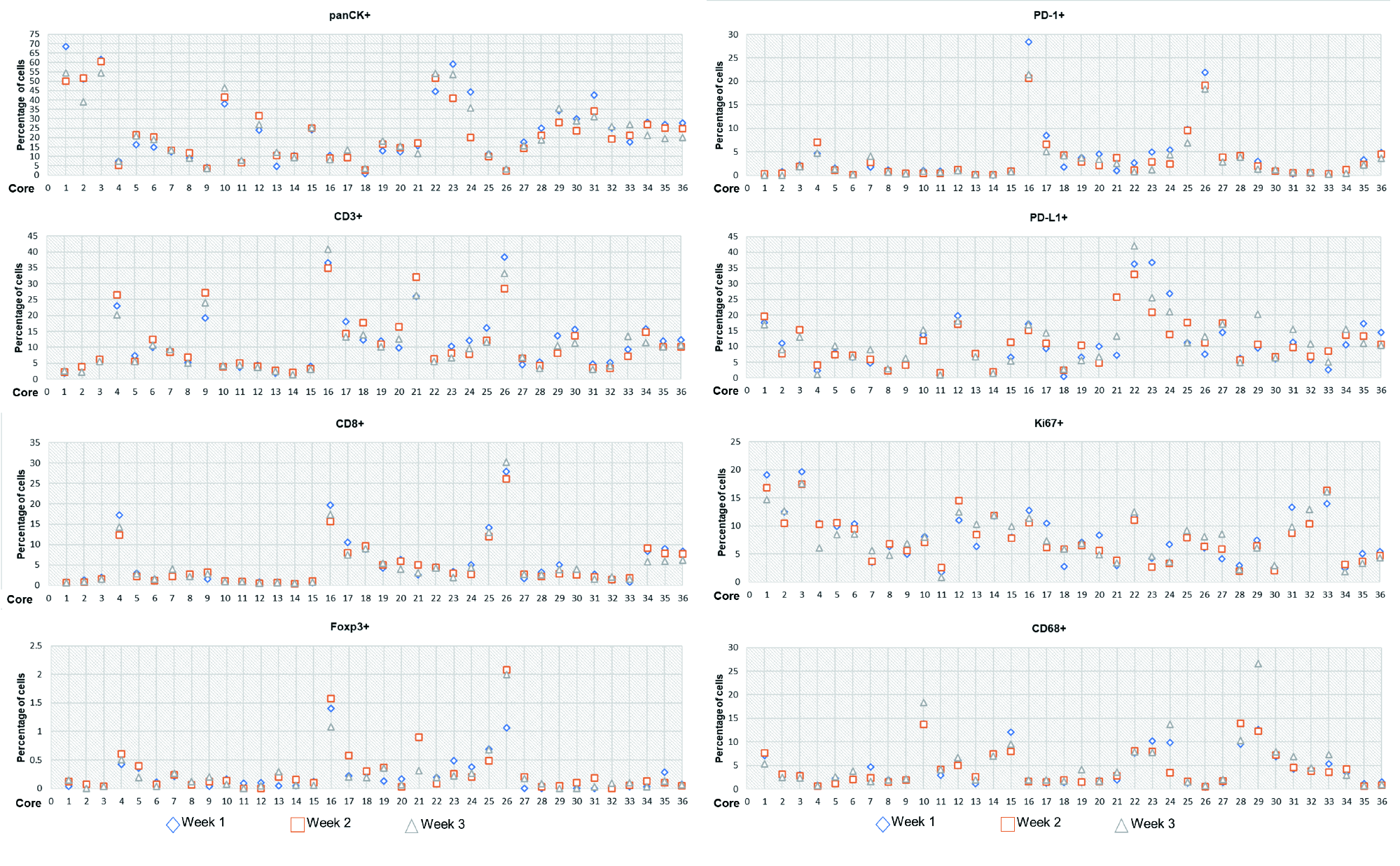

Supplement: Supplementary file 5 — Supplementary Fig. 4. [file 41598_2021_83858_MOESM5_ESM.tif]
