# Supplementary material for: Identification of distinct immune landscapes using an automated nine-color multiplex immunofluorescence staining panel and image analysis in paraffin tumor tissues
Source: Sci Rep. 2021 Feb 25;11:4530. doi: 10.1038/s41598-021-83858-x (PMC7907283; doi:10.1038/s41598-021-83858-x)
Supplement: Supplementary file 6 — Supplementary Table 1. [file 41598_2021_83858_MOESM6_ESM.docx]

**Identification of distinct complex immune landscapes using a novel automated nine-color multiplex immunofluorescence staining panel and image analysis in paraffin tumor tissues**

Edwin R. Parra, Jie Zhai, Auriole Tamegnon, Nicolas Zhou, Renganayaki Krishna Pandurengan, Carmelia Barreto, Mei Jiang, David C. Rice, Caitlin Creasy, Ara A. Vaporciyan, Wayne L. Hofstetter, Anne S. Tsao, Ignacio I. Wistuba, Boris Sepesi, Cara Haymaker.

**Supplementary Table 1.** Clinicopathologic characteristics from patients with malignant pleural mesothelioma (N=12).

| **Variable** | **Category** | **Number (%)** |
| --- | --- | --- |
| Age at Diagnosis | Median (Range) | 66.5 (59-74) years old |
| Gender | Male | 7 (58.3%) |
|  | Female | 5 (41.7%) |
| Smoking history | No | 7 (58.3%) |
|  | Yes | 5 (41.7%) |
| Asbestos exposure | No | 1 (8.3%) |
|  | Yes | 4 (33.3%) |
|  | Unknown | 7 (58.4%) |
| Tumor status (AJCC8)* | pT1 | 4 (33.3%) |
|  | pT2 | 3 (25.0%) |
|  | pT3 | 4 (33.3%) |
|  | pT4 | 1 (8.3%) |
| Nodal status (AJCC8)* | pN0 | 8 (66.7%) |
|  | pN1 | 4 (33.3%) |
| Stage (AJCC8)* | 1A | 3 (25.0%) |
|  | 1B | 3 (25.0%) |
|  | 2 | 4 (33.3%) |
|  | 3B | 1 (8.3%) |
|  | 4 | 1 (8.3%) |
| Neoadjuvant chemotherapy | No | 0 (0.0%) |
|  | Yes | 12 (100.00%) |
| Adjuvant chemotherapy | No | 6 (50.0%) |
|  | Yes | 6 (50.0%) |
| Adjuvant radiotherapy | No | 7 (58.3%) |
|  | Yes | 5 (41.7%) |
| Recurrence | No | 7 (58.3%) |
|  | Yes | 5 (41.7%) |
| Vital status | Dead | 2 (16.7%) |
|  | Alive | 10 (83.3%) |

*The 8th edition of American Joint Committee on Cancer (AJCC) staging system.
