# Supplementary material for: Identification of distinct immune landscapes using an automated nine-color multiplex immunofluorescence staining panel and image analysis in paraffin tumor tissues
Source: Sci Rep. 2021 Feb 25;11:4530. doi: 10.1038/s41598-021-83858-x (PMC7907283; doi:10.1038/s41598-021-83858-x)
Supplement: Supplementary file 7 — Supplementary Table 2. [file 41598_2021_83858_MOESM7_ESM.docx]

**Supplementary Table 2.** Antibody optimization by multiplex immunofluorescence using the Opal fluorophores (Akoya Biosciences).

| **Antibody (Ab)** | **Clone** | **Vendor** | **AR** | **Ab. Dilution** | **F** | **F. Dilution** |
| --- | --- | --- | --- | --- | --- | --- |
| panCK | AE1/AE3 | DAKO | PH6 | 1:100 | 650 | 1:150 |
| CD3 | D7A6E | Cell Signaling | PH6 | 1:100 | 780 | See note |
| CD8 | C8/144B | Thermo Scientific | PH6 | 1:25 | 520 | 1:100 |
| FOXP3 | D2W8E | Cell Signaling | PH6 | 1:50 | 570 | 1:100 |
| KI67 | MIB-1 | DAKO | PH9 | 1:100 | 480 | 1:100 |
| PD-1 | [EPR4877(2)] | ABCAM | PH9 | 1:250 | 620 | 1:100 |
| PD-L1 | E1L3N | Cell Signaling | PH6 | 1:1500 | 690 | 1:150 |
| CD68 | PG-M1 | DAKO | PH6 | 1:50 | 540 | 1:100 |

AR, antigen retrieval; panCK, pancytokeratin; Ab, antibody dilution; F, fluorophore.

The Opal Polaris 780 Fluorophore is a two parts reaction and contains opal TSA-DIG (1:100) and Opal Polaris 780 (1:25)
