# Supplementary material for: Identification of distinct immune landscapes using an automated nine-color multiplex immunofluorescence staining panel and image analysis in paraffin tumor tissues
Source: Sci Rep. 2021 Feb 25;11:4530. doi: 10.1038/s41598-021-83858-x (PMC7907283; doi:10.1038/s41598-021-83858-x)
Supplement: Supplementary file 8 — Supplementary Table 3. [file 41598_2021_83858_MOESM8_ESM.docx]

**Supplementary Table 3.** Correlations of the three time points of staining.

| **Marker** | **Rho and *P*-value** | **Time Points of Staining** | | |
| --- | --- | --- | --- | --- |
|  |  | **week 1 *vs* week 2** | **week 1 *vs* week 3** | **week 2 *vs* week 3** |
| panCK | Rho | 0.932 | 0.951 | 0.938 |
|  | *P* | >0.001 | >0.001 | >0.001 |
| CD3 | Rho | 0.926 | 0.921 | 0.956 |
|  | *P* | >0.001 | >0.001 | >0.001 |
| CD8 | Rho | 0.928 | 0.940 | 0.951 |
|  | *P* | >0.001 | >0.001 | >0.001 |
| FOXP3 | Rho | 0.628 | 0.662 | 0.761 |
|  | *P* | >0.001 | >0.001 | >0.001 |
| PD-1 | Rho | 0.910 | 0.899 | 0.938 |
|  | *P* | >0.001 | >0.001 | >0.001 |
| PD-L1 | Rho | 0.835 | 0.863 | 0.830 |
|  | *P* | >0.001 | >0.001 | >0.001 |
| KI67 | Rho | 0.900 | 0.840 | 0.918 |
|  | *P* | >0.001 | >0.001 | >0.001 |
| CD68 | Rho | 0.892 | 0.883 | 0.865 |
|  | *P* | >0.001 | >0.001 | >0.001 |

**Note:** panCK, pancytokeratin.
